# Folded Heterogeneous Silicon and Lithium Niobate Mach–Zehnder Modulators with Low Drive Voltage

**DOI:** 10.3390/mi12070823

**Published:** 2021-07-14

**Authors:** Shihao Sun, Mengyue Xu, Mingbo He, Shengqian Gao, Xian Zhang, Lidan Zhou, Lin Liu, Siyuan Yu, Xinlun Cai

**Affiliations:** State Key Laboratory of Optoelectronic Materials and Technologies, School of Electronics and Information Technology, Sun Yat-sen University, Guangzhou 510000, China; sunshh6@mail2.sysu.edu.cn (S.S.); xumy26@mail2.sysu.edu.cn (M.X.); hemingb@mail2.sysu.edu.cn (M.H.); gaoshq7@mail2.sysu.edu.cn (S.G.); zhangx733@mail2.sysu.edu.cn (X.Z.); zhould@mail2.sysu.edu.cn (L.Z.); liulin25@mail.sysu.edu.cn (L.L.); yusy@mail.sysu.edu.cn (S.Y.)

**Keywords:** integrated photonics, electro-optical modulator, thin-film lithium niobate, optical communication

## Abstract

Optical modulators were, are, and will continue to be the underpinning devices for optical transceivers at all levels of the optical networks. Recently, heterogeneously integrated silicon and lithium niobate (Si/LN) optical modulators have demonstrated attractive overall performance in terms of optical loss, drive voltage, and modulation bandwidth. However, due to the moderate Pockels coefficient of lithium niobate, the device length of the Si/LN modulator is still relatively long for low-drive-voltage operation. Here, we report a folded Si/LN Mach–Zehnder modulator consisting of meandering optical waveguides and meandering microwave transmission lines, whose device length is approximately two-fifths of the unfolded counterpart while maintaining the overall performance. The present devices feature a low half-wave voltage of 1.24 V, support data rates up to 128 gigabits per second, and show a device length of less than 9 mm.

## 1. Introduction

By harnessing the mature CMOS foundries, silicon photonics based on silicon-on-insulator (SOI) platforms promises the low cost and high volume production for photonic integrated circuits (PICs) [1,2]. This makes it a leading technology for future optical transceivers in both short-reach and long-haul optical communication links, in which high-speed, low-drive-voltage, and low-loss modulators are crucial components [3,4]. Conventional silicon optical modulators rely mainly on the free-carrier effect in silicon p–n junctions. Unfortunately, the free-carrier effect has intrinsic limitations in modulation bandwidth, optical loss and nonlinear response [5,6,7,8,9]. Indeed, the current performance metrics of the silicon optical modulator are more or less close to its physical limits, but future applications require even higher performance due to the insatiable demands of ever-increasing data capacity.

Tremendous efforts have been made toward heterogeneously integrating materials with strong electro-optic effects onto the silicon photonics platform, including graphene [10], electro-optic (EO) polymers [11], indium phosphide (InP) [12], and barium titanate (BTO) [13]. In addition to these materials, lithium niobate (LN), the most successful material for EO modulation [14,15,16], has also been integrated with silicon photonic circuits by using die-to-wafer bonding technique [17,18,19,20]. To date, the heterogeneous LN/SOI Mach–Zehnder modulators (MZMs) have exhibit very appealing overall performance in terms of half-wave voltage (V_π_), modulation bandwidth, and optical loss. However, the length of the Si/LN MZM is relatively long for low-drive-voltage operation, due to the moderate modulation efficiency (V_π_L). The “holy grail” of the optical modulator research is a fast and low-loss device with a V_π_ of about 1 V, which would make it possible to drive the device directly by CMOS electronic circuits and would significantly reduce the cost and power consumption of optical transceivers. For Si/LN MZM, the modulation efficiency is about 2–3 V∙cm. Therefore, the device length needs to be 20 mm or more to obtain a V_π_ of about 1 V. Such a slender structure brings inconvenience to integration with other devices to form sophisticated photonic integrated circuits, and it is difficult to adapt to a compact transceiver package, such as QSFP-DD (quad small form factor pluggable double density).

Here, we report a folded Si/LN traveling wave MZM, in which the optical arms of MZM and traveling wave electrode (TWE) each undergo two 180-degree U-turns, thereby reducing the length of the phase modulation region by nearly 60%. Moreover, by matching the time delays of the optical and microwave U-turns, a perfect velocity match between the optical wave and the modulating microwave can be achieved over the entire modulation section, leading to a large modulation bandwidth. For a folded Si/LN MZM with device length of less than 9 mm, we measure a V_π_ as low as 1.24 V. Such a device can be driven directly by low-cost and energy-efficient CMOS electronic circuits. In addition, the present device shows a low on-chip insertion loss of <3 dB and large extinction ratio of >35 dB. On–off keying (OOK) modulation up to 100 Gbit/s and PAM-4 modulation up to 128 Gbit/s are successfully demonstrated.

## 2. Materials and Methods

**Figure 1:**
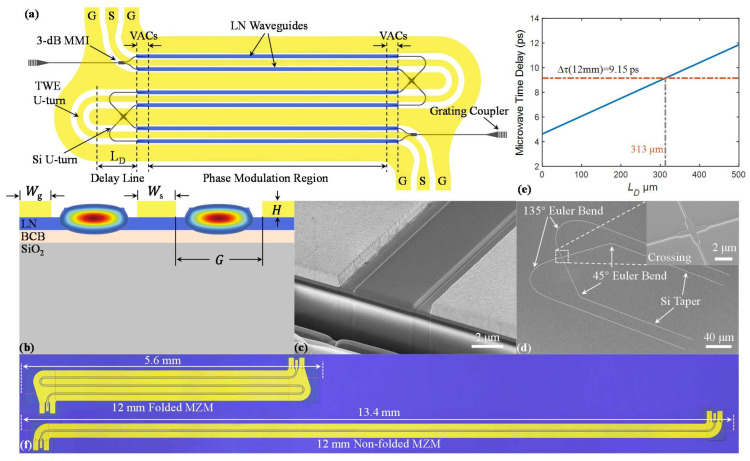
(**a**) Schematic of the folded Si/LN MZM. (**b**) Cross-sectional view of the phase shifter. (**c**) Scanning electron microscopy (SEM) image of the cross-section of the phase modulation region. (**d**) SEM image of the Si U-turn waveguides and crossing. (**e**) Microwave time delay as a function of the length of delay line (L_D_). (**f**) Optical image of the 12 mm folded and unfolded MZMs, the actual device lengths including coupling regions (≈200 μm per side) are 5.6 mm and 13.4 mm, respectively.

Figure 1a shows the schematics of the present folded Si/LN MZM. The device consists of an SOI substrate (220 nm silicon, 3 μm buried oxide layer) with a 600 nm thick layer of X-cut LN membranes on top fabricated based on benzocyclobuten (BCB) adhesive die-to-wafer bonding technology. The top layer is an array of LN waveguides for pure phase modulation (through the Pockels effect). The bottom silicon photonic waveguide supports all the other passive functions, consisting of grating couplers for off-chip coupling, 3 dB multimode interference (MMI) couplers that split and combine the optical power, as well as bent and cross waveguides for folding the optical structure. Optical wave couples up and down between the two layers via vertical adiabatic couplers (VACs), formed by silicon inverse tapers and overlapping LN waveguides.

The meandering arms of the MZM consist of three parallel straight phase modulation sections interconnected by two optical and microwave U-turns. The straight phase modulation section is composed of a pair of LN waveguides running through the gaps of the ground–signal–ground (GSG) TWE (Figure 1b), which operates in a single-drive push–pull configuration. As a result, the applied microwave fields induce a positive phase shift in one LN waveguide and a negative phase shift in the other, leading to nearly chirp-free modulation. The optical waves are coupled from one phase modulation section to another, by passing successively through a pair of VACs, a pair of mutually crossed silicon U-turn waveguides, and another pair of VACs. Here, the two silicon U-turn waveguides were deliberately designed to be mutually crossed. This is because the direction of the electric field is flipped after folding the TWE. To avoid the phase difference in the LN arms being cancelled out before and after the U-turn, the silicon waveguides need to be crossed to ensure that phase differences continue to accumulate. All silicon U-turn waveguides are located at the bottom of Au electrodes, and the interlayers consist of BCB (300 nm) and LN (600 nm), which can promise a negligible absorption loss of silicon waveguide.

We elaborate the design of the U-turns in both optical waveguides and TWE, which are the most critical part of the device and have to be carefully designed in order to provide velocity matching and to minimize extra microwave and optical losses. The detailed designs of the other parts, including a straight LN waveguide, straight TWE, 3dB MMI, and VACs, can be found in Ref [19]. As shown in Figure 1d, the silicon U-turn optical waveguides consist of three parts. The first part is a 135-degree Euler bend with a minimum curvature radius of 28 µm. The second part is a waveguide cross [21] with a low insertion loss of <0.15 dB and low crosstalk of < −40 dB (Supplementary Materials, Section S1). The third part is a 45-degree Euler bend with a minimum curvature radius of 15 µm. Due to the high index contrast of the silicon waveguide, the minimum curvature radius for Euler bends can be less than 5 μm. The reason we choose moderate radius values here is to ensure that the middle ground is wide enough to avoid unwanted microwave crosstalk (Supplementary Materials, Section S2). In this work, the widths of the signal and ground electrodes are set to 17 µm and 120 µm, while the thickness and gap are set to 800 nm and 7.3 µm, respectively, to achieve good impedance and optical-microwave velocity matches in the modulation region. The TWE U-turn consists of two microwave delay lines and a 180-degree circular bent TWE, as shown in Figure 1a. The radius of the circular bent TWE is 75.8 µm measured at the center of the S electrode. The simulation results indicate that such a bent electrode features a low microwave loss and shows a low microwave reflection at the interface between the bent and straight electrodes (Supplementary Materials, Section S2). The microwave delay line is another ingredient needed to be carefully designed for large modulation bandwidth. Due to the large group index of the silicon waveguide (≈4.0), the optical waves slow down considerably after coupling to the silicon optical U-turn. The velocity mismatch between optical and microwave waves in their respective U-turns can be compensated by choosing a proper length for the microwave delay line. We first calculated the time delay of the optical wave including silicon optical U-turn, VAC, and the mismatch in modulation region, with obtained result of ∆τ = 9.15 ps. Figure 1e shows the calculated time delay for microwave delay lines at different lengths (the details of calculation can be found in Supplementary Materials, Section S3). The simulation result indicate that the time delay of optical and microwave U-turns can be matched for a microwave delay line of L_D_ = 313 μm, corresponding to the actual length of 626 μm.

The fabrication process started with patterning silicon waveguides, including 3 dB MMI couplers, inverse tapers for VACs, waveguide crossing, Euler bent waveguides, and grating couplers, using electron beam lithography (EBL) and inductively coupled plasma reactive ion etching (ICP RIE). Next, an X-cut LNOI (from NANOLN) was bonded on top of the fabricated silicon circuits by using BCB die-to-wafer bonding technology. After removing the substrate and the buried oxide layer of the LNOI die, the patterns of LN waveguides were defined by EBL formed by the dry etching process in an Ar^+^ plasma. Finally, the gold TWEs were fabricated by optical lithography, electron beam evaporation, and the lift-off process.

## 3. Results and Discussions

**Figure 2:**
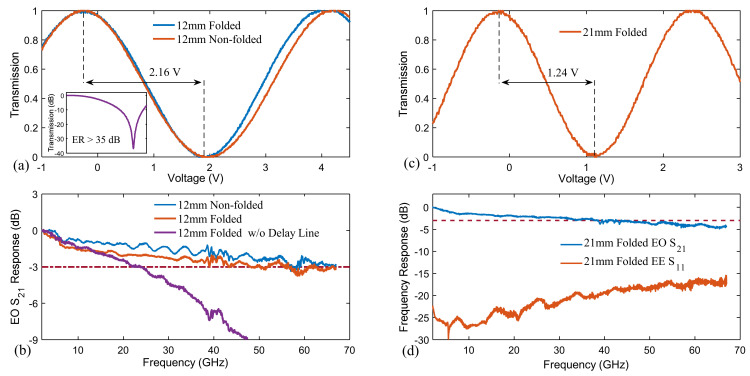
(**a**) Normalized optical transmissions of 12 mm folded and non-folded MZMs as a function of the applied voltage, showing V_π_ of 2.16 V and 2.25 V, respectively. The inset shows the measured normalized transmission on a logarithmic scale. (**b**) Measured EO S_21_ responses of 12 mm non-folded MZM, 12 mm folded MZM, and 12 mm folded MZM without microwave delay line. (**c**) Normalized optical transmission of 21 mm folded MZM as a function of the applied voltage, showing V_π_ of 1.24 V. (**d**) Measured EO S_21_ and RF S_11_ response of 21 mm folded MZM.

To validate the folded MZM design, we fabricated and measured folded devices with a total modulation length of 12 mm (Figure 1f). For comparison, the non-folded counterpart was also fabricated on the same wafer. Due to the two U-turns, the length of the folded device is reduced to approximately two-fifths that of the non-folded device, which is about 5.6 mm. We first employed the 100 kHz triangular voltage sweep method to characterize the V_π_ for both devices. The V_π_ values for the folded and non-folded devices are 2.16 V and 2.25 V, corresponding to voltage–length products of 2.6 V∙cm and 2.7 V∙cm, respectively. The slightly lower V_π_ value for the folded device is because of the additional phase modulation in VACs at both ends of the optical U-turn waveguides. The inset of Figure 2a shows the transmission of the folded device, demonstrating a measured extinction ratio of >35 dB. Next, we characterized the EO bandwidth (S_21_ parameter) for three types of devices: (1) non-folded device, (2) folded device with velocity-matched microwave delay lines (L_D_ = 313 μm), and (3) folded device without microwave delay lines (L_D_ = 0), as depicted in Figure 2b. The measured modulation bandwidth of the folded and non-folded MZM is 50 GHz and 60 GHz (with a reference frequency of 2 GHz), respectively. The bandwidth of the folded device with velocity-matched microwave delay lines is slightly smaller than the non-folded device because the TWE in the folded device possesses extra microwave loss due to the additional U-turns. In contrast, bandwidth of the folded device without microwave delay lines is significantly smaller than the other cases. These results show that compensating for the optical wave and microwave time delay is essential to achieve large bandwidths in folded MZMs.

To further reduce the V_π_, we fabricated folded devices with a total modulation length of 21 mm, whose actual device length is 8.7 mm. As shown in Figure 2c,d, the measured V_π_ and bandwidth for such a device is 1.24 V and 40 GHz, respectively, which are very well facilitated for high-speed and low-drive-voltage operation. The RF return losses (S_11_ parameter) of the devices are less than—15 dB at up to 67 GHz, indicating that the microwave U-turns do not introduce significant impedance discontinuities in TWE. The device also features a low on-chip loss of 2.9 dB.

**Figure 3:**
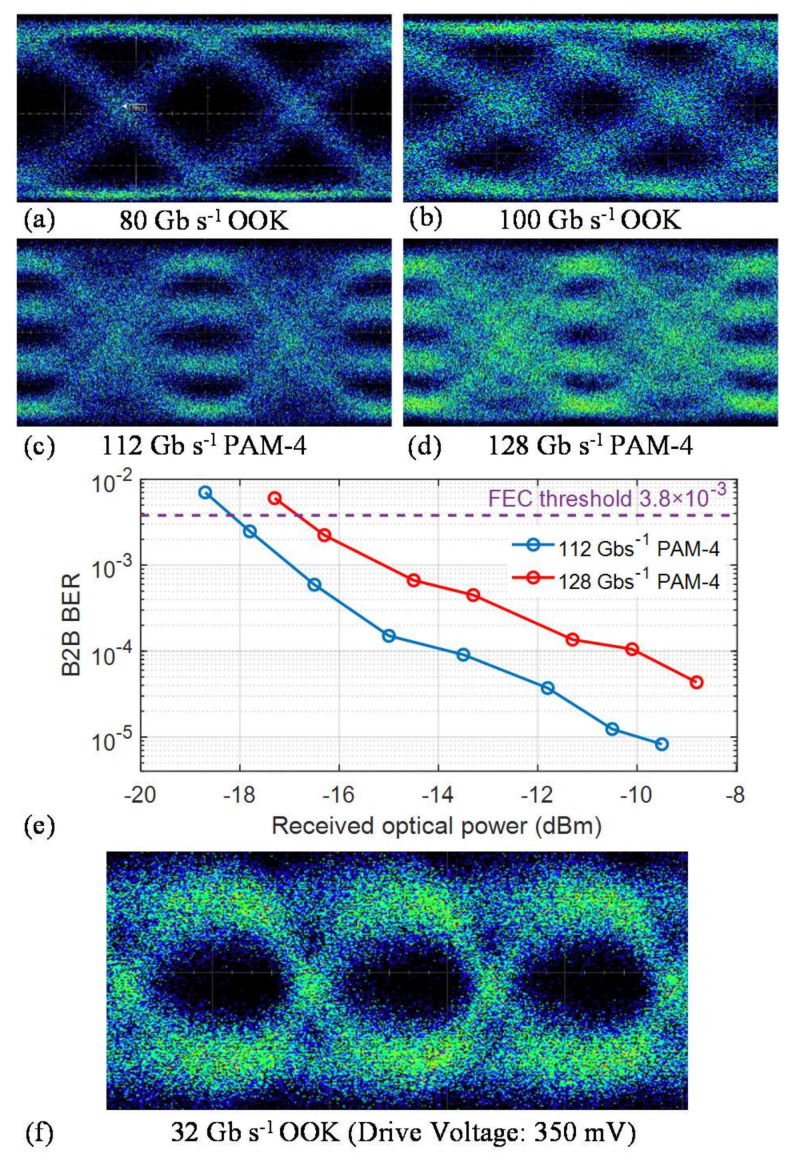
High-speed transmission experiments. (**a**,**b**) Measured optical eye diagrams for OOK modulation at date rates of 84 Gb s^−1^ and 100 Gb s^−1^. (**c**,**d**) Measured optical eye diagrams for PAM-4 modulation at data rates of 56 Gbaud (112 Gb s^−1^) and 64 Gbaud (128 Gb s^−1^). (**e**) Measured back-to-back BER curves versus the received optical power for 56 Gbaud (112 Gb s^−1^) and 64 Gbaud (128 Gb s^−1^) PAM-4 signal. (**f**) Measured optical eye diagram for OOK signal at date rate of 32 Gb s^−1^ without any RF amplifier.

The high-speed modulation ability of the modulator has also been characterized. First, we use the 12 mm device to demonstrate high-speed data modulation. Figure 3a,b show the optical eye diagrams for OOK modulation at 80 Gb s^−1^ and 100 Gb s^−1^. The measured extinction ratios are 7.8 and 5.8 dB, respectively. We also performed the PAM-4 modulation experiments at 56 Gbaud (112 Gb s^−1^) and 64 Gbaud (128 Gb s^−1^); the results are shown in Figure 3c,d. Figure 3e shows the back to back (B2B) bit-error rate (BER) versus the received optical power for 112 Gb s^−1^ and 128 Gb s^−1^ PAM-4 signal transmission. The details of the B2B BER measurements can be found in our previous work [20]. In addition to the data rate difference, the 1.5 dB power penalty between the 56 Gbaud and 64 Gbaud is partly due to the bandwidth constraint of the oscilloscope. Furthermore, we employ a CMOS digital-to-analog converter (DAC) circuit to generate 32 Gbps electrical OOK signals with peak-to-peak voltage of 350 mV, which was used to directly drive the fabricated low-V_π_ modulator with the total modulation length of 21 mm. Figure 3f shows the optical eye diagram with measured extinction ratio of 2.7 dB. We also performed higher data rate experiments up to 112 Gb s^−1^ with an RF amplifier on this device; the results can be found in the Supplementary Materials, Section S4.

## 4. Conclusions

In this paper, we have demonstrated folded MZMs based on a heterogeneous integrated silicon and lithium niobate platform using meandering optical waveguides and TWEs. The devices show reduced device length while maintaining the overall performance (half-wave voltage and bandwidth) of the non-folded counterparts. Since both of the optical waveguides and TWEs undergo two U-turns, the device length is reduced to approximately two-fifths of the original value. A similar folded thin-film lithium niobate modulator had been demonstrated based on the poling process [22]. By employing more U-turns, the length can be future compressed at the cost of increase in width. This architecture provides great design flexibility especially for building sophisticated Si/LN photonic integration circuits, because most silicon photonic devices are very compact in footprint. Moreover, the low-drive-voltage operation demonstrated in the devices could open opportunities not only for future compact and low power consumption optical transceivers but also for the direct integration of CMOS integrated circuits with high-speed modulators and optical switches.

## Data Availability

Not applicable.

## Supplementary Materials

The following are available online at https://www.mdpi.com/article/10.3390/mi12070823/s1. Section S1: Characteristic of Si waveguide crossing, Section S2: Design of microwave U-turns, Section S3: Design of microwave delay line, Section S4: High-speed experiments of 21 mm folded MZM.
