# Supplementary material for: Folded Heterogeneous Silicon and Lithium Niobate Mach–Zehnder Modulators with Low Drive Voltage
Source: Micromachines (Basel). 2021 Jul 14;12(7):823. doi: 10.3390/mi12070823 (PMC8307437; doi:10.3390/mi12070823)
Supplement: Supplementary file 1 [file micromachines-12-00823-s001.zip › micromachines-1289409-supplementary.pdf]

Folded Heterogeneous Silicon and Lithium Niobate Mach-Zehnder Modulators with Low Drive Voltage: Supplementary Material

S1. Characteristic of Si Waveguide Crossing

Since the waveguide crossings are responsible for connecting silicon waveguides and exchanging the two arms in the push-pull modulator, the optical insertion loss, crosstalk, and the size of the silicon crossing are essential. In this work, we design the crossing based on photonics inverse design. Figure S1a shows the simulated electric field of the TE0 mode at 1550 nm. The influence of the optimized crossing on the forward light is negligible and almost no light is scattered into the cross waveguide. The footprint of the crossing is ~6.5μm × 6.5μm, which benefits smaller microwave U bend.

To characterize the insertion loss and the crosstalk, we fabricated the arrays with a different number of crossings (Figure S1b). Two vertical grating couplers are used for on- and off-chip coupling. Figure S1c shows the measured total insertion loss, including losses from two grating couplers and a crossing. The crosstalk is measured by comparing the transmission of the orthogonal port and the through port. We confirm the crossing has low crosstalk (below -40 dB) at the whole C band. In Figure S1d, we extract the loss per crossing is -0.13 dB at 1550 nm, which amounts to 97% transmission.

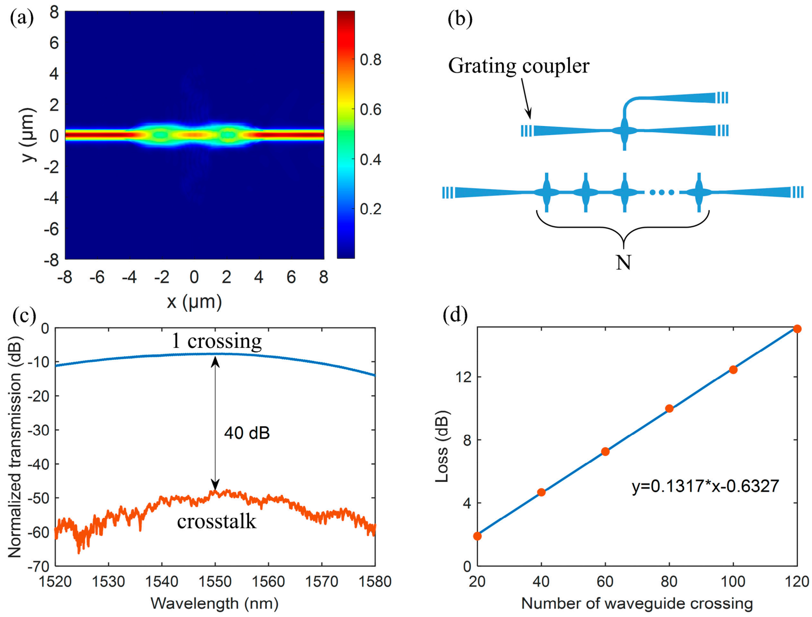

Figure S1. (**a**) Simulated electric field profiles of the silicon waveguide crossing. (**b**) The schematic of the cascaded a series of crossings. (**c**) Measured insertion loss and crosstalk spectra. The results include the insertion loss from a pair of grating couplers. (**d**) Measured insertion loss as a function of the number of crossing at 1550 nm.

S2. Design of Microwave U Turns

It is essential to design the meandering microwave transmission lines with a low RF loss, reflection, and low-crosstalk between parallel coplanar waveguides (CPWs). For modulator application, we desire the negligible coupling between neighboring straight CPW. Generally, the coupling coefficient is closely related to the ground-to-ground spacing d=w+2s and the width of the middle ground w_{mg} [1]. d is determined by the dimension of straight CPW, which is designed to achieve 50-ohm impedance matching, velocity matching between the

microwave and optical wave, and low RF propagation loss. As shown in Figure S2, we investigate the simulated CPW coupling for the different w_{mg}. We observe that the coupling is lower than -40 dB when w_{mg} greater than 120 μm. Therefore, we adopt w_{mg}=120 μm (R= 75.8 μm) in this work for ignorable microwave crosstalk.

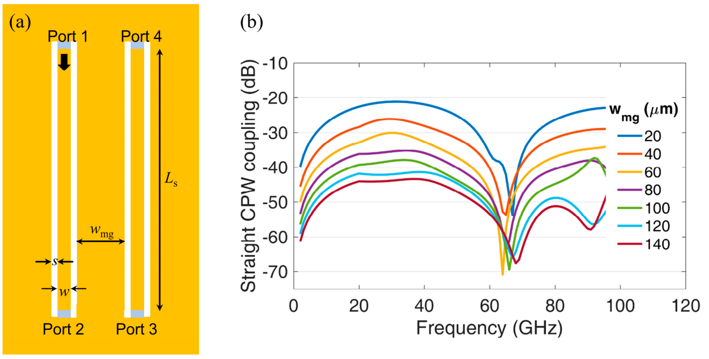

Figure S2. (**a**) Schematic of a 4-port model used to characterize the coupling between parallel CPW. (**b**) Simulated magnitude of transmission coefficienct S_{41} as a function of frequency. L_s=1mm.

The RF loss comes from two sections: the straight CPW and the U-bend CPW. The RF reflection comes from the 50-ohm impedance mismatch of the CPW, and impedance discontinuity connecting the straight and U-bend CPW. We discuss U-turns only in this section. As shown in Figure S3, we display the simulated S_{21} and S_{11} parameters from a U-bend and two short straight CPW. The radius of the circular bent TWE (R=75.8 μm) is the same in the main manuscript. The loss per microwave U bend is only ~- 0.34 dB at 100 GHz. Besides, the S_{11} is below -30 dB until 100 GHz.

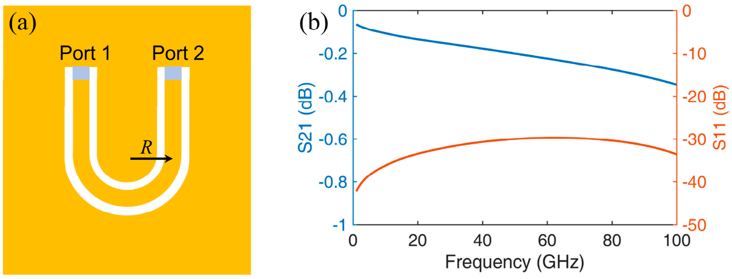

Figure S3. (**a**) Schematic of a 2-port model used to characterize the RF transmission of the U-bend section. (**b**) Simulated magnitude of RF transmission coefficient S_{21} and S_{11} against frequency.

S3. Design of Microwave Delay Line

The EO S_{21} response of folded modulator is determined by the mismatch degree of the optical signal and microwave signal when the structural parameters of travelling wave electrode (TWE) is already confirmed. As seen in Figure S4, we define the initial point at the front of vertical adiabatic coupler (VAC) before Si U-turn, and the end point at the end of the first passing VAC after optical wave propagate direction is turned a 180° rotation. To maximum decrease the delay mismatch between optical and electrical signal, we first calculate the time delay of optical signal. As depicted in Figure S4, for 12 mm folded device, the whole optical delay comes from two VACs ($2\times\tau_{VAC}$), a waveguide crossing (τ_{cross}), and Si waveguides (τ_{Si}) consists of 135° Euler bend of 136.44 μm, 45° Euler bend of 23.56 μm, and the cascaded straight waveguides of 117.24 μm). The group delay of the VAC and crossing devices can be calculated in FDTD product from Lumerical with the results of 2.128 ps and

0.164 ps respectively. Optical mode group velocity of Si waveguide can be confirmed by optical mode solver (Lumerical Mode Solution): $n_{g(Si)}$=4.0476, corresponding to τ_{Si}=3.741 ps. Since the effective index of the TWE at high frequency region is designed as 2.1721, the RF signal delay without any compensate delay line is 4.62 (U-bend electrode of 238μm, two VAC region of 400 μm). So the time delay as a function of the length of delay line L_D can be expressed as:

$$\tau_e = \frac{2n_m}{c} L_D + 4.62 \tag{S1}$$

Where the n_m is the effective index of TWE, the formula can be expressed as the blue curve in Figure S4b. The time delay difference between the optical and RF signal will decrease as the delay line becomes longer, but in fact, the modulation region also exists delay difference due to the velocity mismatch. So, as the optical mode group index of LN waveguide is 2.1721, the total optical time delay of 12mm folded device should be $\Delta\tau=\tau_{Si}+\tau_{cross}+2\times\tau_{VAC}+\tau_{mismatch}$=9.15 ps, and for 21mm folded device this value will be 9.89 ps. As shown in Figure S4(b), the final compensated length of delay line is 313 μm for 12 mm folded device and 364 μm for 21 mm folded device.

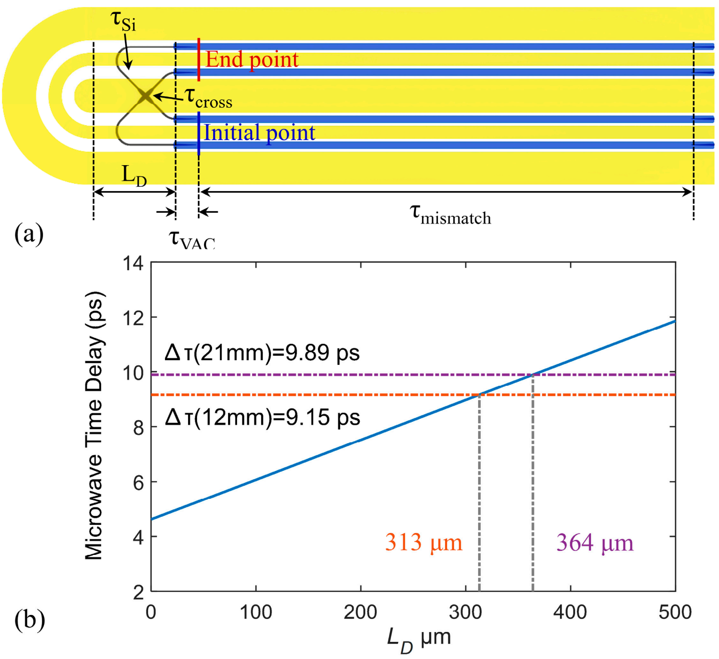

Figure S4. (**a**) Schematic of optical time delay definition. (**b**) Microwave time delay as a function of L_D.

S4. High Speed Experiments of 21 mm Folded MZM

We also performed the high speed OOK and PAM-4 modulation format on 21 mm folded MZM with the same experiment setup as the 12 mm folded MZM (Figure S5a). The RF signal from an arbitrary waveform generator (AWG) is fed to the MZM through a high-speed probe (67 GHz) after amplification (SHF S807). The modulated light is amplified by an erbium-doped fiber amplifier (EDFA) and detected by a high-speed PD connected with a real-time oscilloscope for recording waveforms. Figure S5b,c shows the optical eye diagrams for OOK modulation at 56 Gb s^{-1} and 80 Gb s^{-1}. The measured extinction ratios are 10.2 and 6.9 dB respectively. We also performed 40 Gbaud (80 Gb s^{-1}) and 56 Gbaud (112 Gb s^{-1}) PAM-4

modulation format on the modulator, and the optical eye diagrams are depicted in Figure S5d,e.

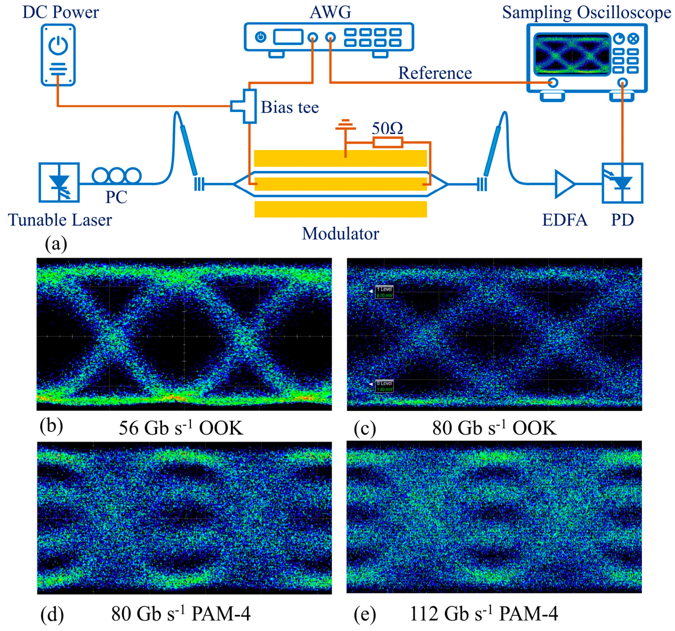

Figure S5. (**a**) Experimental set-up for measuring the eye diagram. AWG: arbitrary waveform generator. (**b**,**c**) Optical eye diagrams for OOK signal at date rate of 56 Gb s^{-1} and 80 Gb s^{-1}. (**d**,**e**) Measured PAM-4 modulation optical eye diagrams at 40 Gbaud (80 Gb s^{-1}) and 56 Gbaud (112 Gb s^{-1}).

Reference

1. Cheng, K.M. Effect of conductor backing on the line-to-line coupling between parallel coplanar lines. *IEEE Trans. Microwave Theory Tech.* **1997**, *45*, 1132–1134.
